# Deep learning analysis of clinical course of primary nephrotic syndrome: Japan Nephrotic Syndrome Cohort Study (JNSCS)

**DOI:** 10.1007/s10157-022-02256-3

**Published:** 2022-08-12

**Authors:** Tomonori Kimura, Ryohei Yamamoto, Mitsuaki Yoshino, Ryuichi Sakate, Enyu Imai, Shoichi Maruyama, Hitoshi Yokoyama, Hitoshi Sugiyama, Kosaku Nitta, Tatsuo Tsukamoto, Shunya Uchida, Asami Takeda, Toshinobu Sato, Takashi Wada, Hiroki Hayashi, Yasuhiro Akai, Megumu Fukunaga, Kazuhiko Tsuruya, Kosuke Masutani, Tsuneo Konta, Tatsuya Shoji, Takeyuki Hiramatsu, Shunsuke Goto, Hirofumi Tamai, Saori Nishio, Kojiro Nagai, Kunihiro Yamagata, Hideo Yasuda, Shizunori Ichida, Tomohiko Naruse, Tomoya Nishino, Hiroshi Sobajima, Toshiyuki Akahori, Takafumi Ito, Yoshio Terada, Ritsuko Katafuchi, Shouichi Fujimoto, Hirokazu Okada, Tetsushi Mimura, Satoshi Suzuki, Yosuke Saka, Tadashi Sofue, Kiyoki Kitagawa, Yoshiro Fujita, Makoto Mizutani, Naoki Kashihara, Hiroshi Sato, Ichiei Narita, Yoshitaka Isaka

**Affiliations:** 1 Reverse Translational Research Project, Center for Rare Disease Research, National Institutes of Biomedical Innovation, Health and Nutrition (NIBIOHN), Ibaraki, Osaka Japan; 2 Laboratory of Rare Disease Resource Library, Center for Rare Disease Research, National Institutes of Biomedical Innovation, Health and Nutrition (NIBIOHN), Ibaraki, Osaka Japan; 3 Health and Counseling Center, Osaka University, Suita, Osaka Japan; 4 Nakayamadera Imai Clinic, Takarazuka, Hyogo Japan; 5 Department of Nephrology, Nagoya University Graduate School of Medicine, Nagoya, Aichi Japan; 6 Department of Nephrology, Kanazawa Medical University School of Medicine, Kanazawa, Japan; 7 Department of Nephrology, Rheumatology, Endocrinology and Metabolism, Okayama University Graduate School of Medicine, Dentistry and Pharmaceutical Sciences, Okayama, Japan; 8 Department of Nephrology, Tokyo Women’s Medical University, Tokyo, Japan; 9 Department of Nephrology and Dialysis, Kitano Hospital, Tazuke Kofukai Medical Research Institute, Osaka, Japan; 10 Department of Internal Medicine, Teikyo University School of Medicine, Tokyo, Japan; 11 Kidney Disease Center, Japanese Red Cross Nagoya Daini Hospital, Nagoya, Aichi Japan; 12 Department of Nephrology, JCHO Sendai Hospital, Sendai, Miyagi Japan; 13 Department of Nephrology and Laboratory Medicine, Kanazawa University, Kanazawa, Japan; 14 Department of Nephrology, Fujita Health University School of Medicine, Toyoake, Aichi Japan; 15 First Department of Internal Medicine, Nara Medical University, Nara, Japan; 16 Division of Nephrology, Department of Internal Medicine, Toyonaka Municipal Hospital, Toyonaka, Osaka Japan; 17 Department of Nephrology, Nara Medical University, Kashihara, Nara Japan; 18 Division of Nephrology and Rheumatology, Department of Internal Medicine, Faculty of Medicine, Fukuoka University, Fukuoka, Japan; 19 Department of Cardiology, Pulmonology, and Nephrology, Yamagata University School of Medicine, Yamagata, Japan; 20 Department of Kidney Disease and Hypertension, Osaka General Medical Center, Osaka, Japan; 21 Department of Nephrology, Konan Kosei Hospital, Konan, Aichi Japan; 22 Division of Nephrology and Kidney Center, Kobe University Graduate School of Medicine, Kobe, Hyogo Japan; 23 Department of Nephrology, Anjo Kosei hospital, Anjo, Aichi Japan; 24 Division of Rheumatology, Endocrinology and Nephrology, Hokkaido University Graduate School of Medicine, Sapporo, Japan; 25 Department of Nephrology, Institute of Biomedical Sciences, Tokushima University Graduate School, Tokushima, Japan; 26 Department of Nephrology, Faculty of Medicine, University of Tsukuba, Tsukuba, Ibaragi Japan; 27 Internal Medicine 1, Hamamatsu University School of Medicine, Hamamatsu, Shizuoka Japan; 28 Department of Nephrology, Japanese Red Cross Nagoya Daiichi Hospital, Nagoya, Aichi Japan; 29 Department of Nephrology, Kasugai Municipal Hospital, Kasugai, Aichi Japan; 30 Department of Nephrology, Nagasaki University Hospital, Nagasaki, Japan; 31 Department of Diabetology and Nephrology, Ogaki Municipal Hospital, Ogaki, Ogagki Japan; 32 Department of Nephrology, Chutoen General Medical Center, Kakegawa, Shizuoka Japan; 33 Division of Nephrology, Shimane University Hospital, Izumo, Shimane Japan; 34 Department of Endocrinology, Metabolism and Nephrology, Kochi Medical School, Kochi University, Kochi, Japan; 35 Kideny Unit, National Hospital Organization, Fukuoka-Higashi Medical Center, Koga, Fukuoka Japan; 36 Department of Hemovascular Medicine and Artificial Organs, Faculty of Medicine, University of Miyazaki, Miyazaki, Japan; 37 Department of Nephrology, Saitama Medical University, Iruma, Saitama Japan; 38 Department of Nephrology, Gifu Prefectural Tajimi Hospital, Tajimi, Gifu Japan; 39 Department of Nephrology, Kainan Hospital, Yatomi, Aichi Japan; 40 Department of Nephrology, Yokkaichi Municipal Hospital, Yokkaichi, Mie Japan; 41 Department of Cardiorenal and Cerebrovascular Medicine, Kagawa University, Takamatsu, Kagawa Japan; 42 Division of Internal Medicine, National Hospital Organization Kanazawa Medical Center, Kahoku, Kanazawa Japan; 43 Department of Nephrology, Chubu Rosai Hospital, Nagoya, Aichi Japan; 44 Department of Nephrology, Handa City Hospital, Handa, Aichi Japan; 45 Department of Nephrology and Hypertension, Kawasaki Medical School, Kurashiki, Okayama Japan; 46 Department of Nephrology, Endocrinology, and Vascular Medicine, Tohoku University Graduate School of Medicine, Sendai, Miyagi Japan; 47 Division of Clinical Nephrology and Rheumatology, Kidney Research Center, School of Medical and Dental Sciences, Niigata University Graduate, Niigata, Japan; 48 Department of Nephrology, Osaka University Graduate School of Medicine, Suita, Osaka Japan

**Keywords:** Nephrotic syndrome, Machine learning, Clinical course, Prognosis, Proteinuria, Creatinine, Hematuria

## Abstract

**Background:**

Prognosis of nephrotic syndrome has been evaluated based on pathological diagnosis, whereas its clinical course is monitored using objective items and the treatment strategy is largely the same. We examined whether the entire natural history of nephrotic syndrome could be evaluated using objective common clinical items.

**Methods:**

Machine learning clustering was performed on 205 cases from the Japan Nephrotic Syndrome Cohort Study, whose clinical parameters, serum creatinine, serum albumin, dipstick hematuria, and proteinuria were traceable after kidney biopsy at 5 measured points up to 2 years. The clinical patterns of time-series data were learned using long short-term memory (LSTM)-encoder–decoder architecture, an unsupervised machine learning classifier. Clinical clusters were defined as Gaussian mixture distributions in a two-dimensional scatter plot based on the highest log-likelihood.

**Results:**

Time-series data of nephrotic syndrome were classified into four clusters. Patients in the fourth cluster showed the increase in serum creatinine in the later part of the follow-up period. Patients in both the third and fourth clusters were initially high in both hematuria and proteinuria, whereas a lack of decline in the urinary protein level preceded the worsening of kidney function in fourth cluster. The original diseases of fourth cluster included all the disease studied in this cohort.

**Conclusions:**

Four kinds of clinical courses were identified in nephrotic syndrome. This classified clinical course may help objectively grasp the actual condition or treatment resistance of individual patients with nephrotic syndrome.

**Supplementary Information:**

The online version contains supplementary material available at 10.1007/s10157-022-02256-3.

## Introduction

Nephrotic syndrome is characteristic by massive proteinuria, edema, and hypoalbuminemia [1]. Nephrotic syndrome is often poor in prognosis and complicated with a wide variety of adverse events, including end-stage kidney disease (ESKD) [2, 3], thromboembolism [4], infection [5], malignancy [6], cardiovascular disease (CVD) [7], and mortality [8]. Primary nephrotic syndrome is the major cause of nephrotic syndrome, which includes minimal change disease (MCD), membranous nephropathy (MN), and focal segmental glomerulosclerosis (FSGS) [9]. The reported incidences of MCD, MN, and FSGS were 0.2–0.8, 0.3–1.4, and 0.2–1.1 per 100,000 person-years, respectively [10] .

The overall prognosis of nephrotic syndrome has not been evaluated based on objective clinical items. Currently, key clinical information and decisions, such as prediction of the prognosis and determination of treatment strategy, rely on kidney biopsy, and pathological diagnosis has difficulty in eliminating subjectivity. The fact that kidney biopsy is rarely performed repeatedly, due to its risk and tendency to be procedurally cumbersome, also suggests that it is preferable to explore objective methods to monitor nephrotic syndrome. Usually, the clinical course of nephrotic syndrome is followed by physiologic findings and laboratory test values. The treatment strategy for nephrotic syndrome is often common and mainly utilizes immunosuppressive therapy [11]. Therefore, objective estimation of the clinical course of nephrotic syndrome provides an opportunity to handle nephrotic syndrome in common and helps in the decision for immunosuppressive therapy.

Nephrotic syndrome is a representative rare and intractable disease. Rare and intractable diseases share common problems in research and development. Smaller patient populations yield limited information on diseases, and multifactorial pathogenesis may result in a complex set of symptoms. It is necessary to overcome this problem to elucidate the natural history of rare and intractable disease, including nephrotic syndrome. Deep learning is a method of artificial intelligence technology that may help to unravel potential patterns of multiple factors. Deep learning methods have been applied in the analysis of clinical patterns of several diseases from complex combinations of clinical parameters. Additionally, an autoencoder (self-encoder), an unsupervised machine learning method, is suitable for learning objectively and without bias, even with a small number of cases [12–15]. A complementary combination of deep learning methods helps to detect clinical patterns of rare and intractable diseases.

In this study, we aimed to mathematically classify the clinical course of nephrotic syndrome based on machine learning algorithms using clinical variables. The Japan Nephrotic Syndrome Cohort Study (JNSCS) aims to clarify the clinical course of nephrotic syndrome [16]. Through machine learning in this cohort, we elucidated the variations in the clinical course of nephrotic syndrome.

## Methods

### Participants

The JNSCS is a multicenter cohort study of primary nephrotic syndrome with a 5-year follow-up period. The main purpose is to elucidate the incidence rates of major clinical outcomes and to assess the effectiveness of immunosuppressive therapy in Japan. Details of the study design were previously described [16]. Briefly, 374 nephrotic patients who were diagnosed with primary nephrotic syndrome by kidney biopsy during the entry period between 2009 and 2010 in 55 hospitals were registered in the JNSCS. The diagnosis of primary nephrotic syndrome was based on the clinical and histopathological characteristics [17]. Nephrotic patients with minor glomerular abnormalities by light microscopy were diagnosed with MCD. The diagnosis of MN was made by the detection of granular deposits of mainly IgG along the glomerular capillary walls by immunofluorescence microscopy with or without thickening of the glomerular capillary wall by light microscopy. FSGS included five variants: collapsing, tip, cellular, perihilar, and not-otherwise specified variants [18]. Other glomerulonephritis included membranoproliferative glomerulonephritis, mesangial proliferative glomerulonephritis, endocapillary proliferative glomerulonephritis, and crescentic glomerulonephritis membranoproliferative glomerulonephritis.

The study protocol of JNSCS was approved by the ethics committee of Osaka University Hospital (approval number 17035–4) and the institutional review board of each participating hospital. All procedures performed in the present study were in accordance with the Declaration of Helsinki.

### Unsupervised machine learning classifier

In this study, the long short-term memory (LSTM)-encoder–decoder architecture, an unsupervised machine learning classifier, was applied. To classify the severity of the clinical course of patients, an existing disease type classification is necessary as the objective variable in supervised machine learning. Since disease classification is generally based on clinical information at the time of initial diagnosis, the predicting of aggravation is not always accurate. Such teacher data itself can form a learning bias and may hinder the objective classification of clinical course variation patterns. The unsupervised machine learning-based time-dependent analysis has the advantage of objective classification of the clinical course.

The first step of this architecture is to apply the LSTM neural network [19]. LSTM learns the fluctuation pattern of time-series data consisting of multiple clinical items based on the relationship between measurement points. LSTM can learn the relationship both before and after the measurement point, as well as the relationship between the values of subsequent measurement points. This makes it possible to characterize and classify clinical courses consisting of clinical items such as multiple values and clinical findings.

The second step is to apply an autoencoder. An autoencoder is a learning model that makes the input and output the same, and is a neural network widely used for dimensional compression and feature extraction. The compressed dimensions can be visualized as a scatter plot with one case as one point if the intermediate layer is two-dimensional or three-dimensional [20]. This intermediate layer is called the feature space, which is considered to reflect the features including the interaction between multiple items. In the feature space, cases with common or similar features form clusters. An autoencoder reduces the entropy of the data distribution, i.e., clutter and noise of information [21]. By reducing the entropy, the data distribution range in the feature space becomes narrower and clusters are more likely to be recognized. This feature of the autoencoder potentiates the gradual reduction of the degree of freedom of the neural network, and is useful for preventing overfitting by reducing the number of weighting factors to learn. Using this characteristic, clinical courses are objectively classified based on item variation patterns. The resultant encoded features represent the time-series variation patterns of clinical items learned by LSTMs. Further information is available at https://github.com/cran2367/understanding-lstm-autoencoder#readme.

### Statistics

For the LSTM-encoder–decoder architecture, the input values of the encoder have 5 dimensions (5 items), followed by the output dimensions of 24 in the first layer of LSTM, 12 in the second fully bonded layer, and 2 in the third layer of LSTM. The decoder restores the input values in the reverse order of the encoder. The mean square error was used for the loss function. Learnings were performed in 5 sets with the conditions of 4 epochs per set, 1,000 learnings per epoch, and a batch size of 10 data points. Learning was not performed in more than the number of epochs where no significant reduction in loss was observed. The learning result was defined by the Gaussian mixture distribution in the cluster in the two-dimensional scatter plot [22]. Each case was assigned to the cluster with the highest log-likelihood. Python version 3.7.5 was used for the analysis. The LSTM-encoder–decoder architecture was built with Torch 1.7.1. The GaussianMixture module of scikit-learn 0.22.2 was used to estimate the parameters of the Gaussian mixture distribution [23]. The specifications of the computer used were 64 bit/Core i9-9900 K/Intel Z370 Express/DDR4-2400 S.O. DIMM (PC4-19,200) 32 GB(16 GB × 2)/GeForce RTX 2080 8 GB GDDR6.

## Results

**Fig. 1.**
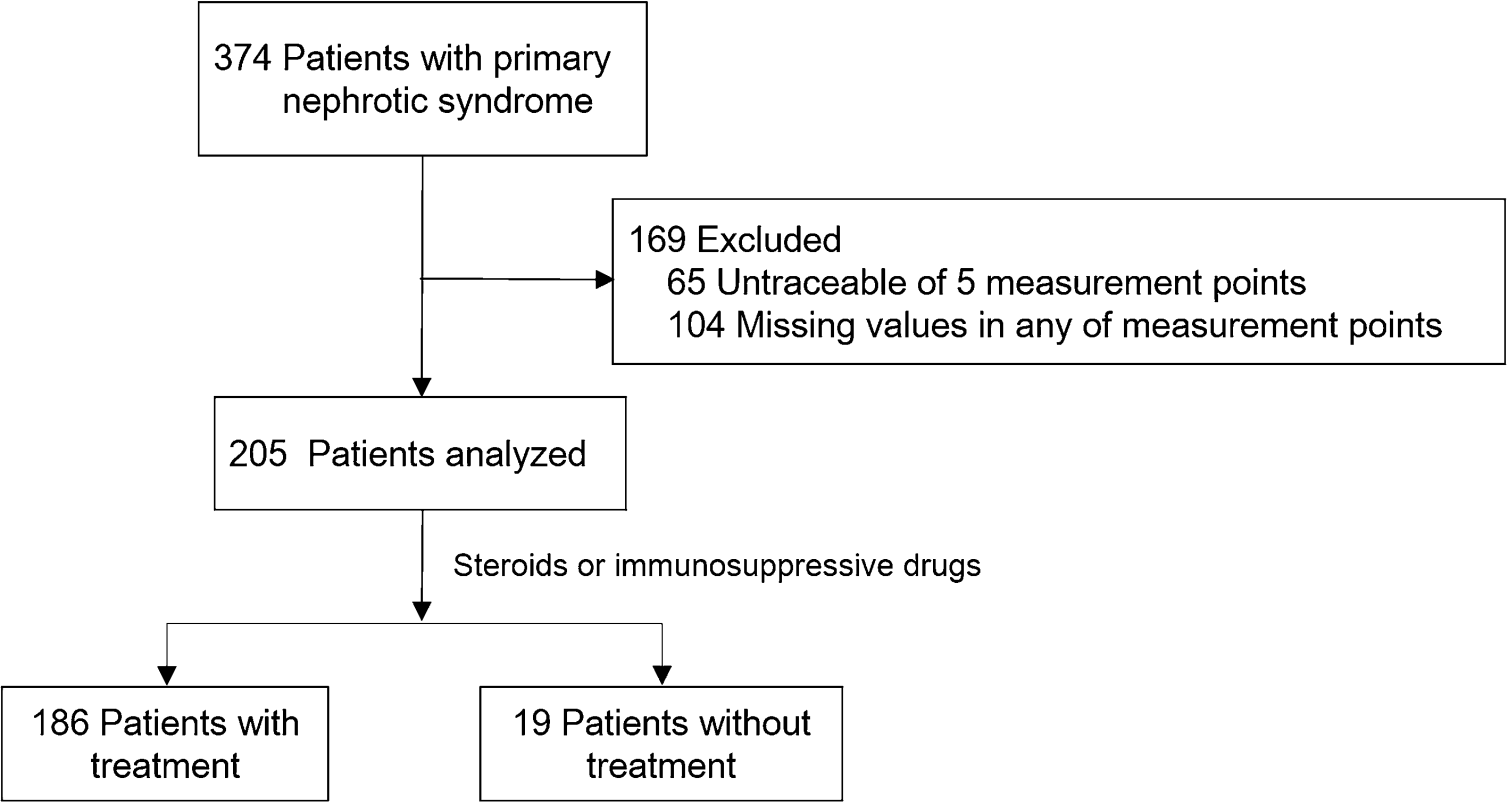
Analyzed patients in this study. The measurement points were five measurement points up to 2 years. Clinical parameters included serum creatinine, serum albumin, qualitative hematuria, qualitative proteinuria, and urine protein per creatinine ratio. Machine learning classification was performed on 205 patients using clinical parameters. Separately, classification was also performed on 186 patients who received steroids or immunosuppressive drugs

The background demographics of the participants are shown in Table 1. Machine learning classification (clustering) was performed on 205 patients, whose clinical parameters were completely traceable at 5 measured points after kidney biopsy for up to 2 years (Fig. 1, Tables 1 and 2, Supplementary Tables S1 and S2). The time-points included 1, 3, 6, 12, and 24 months after kidney biopsy. The participants consisted of 90 (43.9%), 77 (37.6%), 18 (8.8%), and 9 (4.4%) patients with MCD, MN, FGS, and IgA nephropathy, respectively.

**Table 1:** Original kidney diseases for each cluster classified by deep learning

	C1	C2	C3	C4	Total
Age (median (25, 75%))	43.5 (29.0–65.5)	61.0 (47.5–70.5)	66.0 (45.0–75.0)	65.0 (58.0–75.0)	58.0 (38.0–72.0)
Sex (man, woman)	44–41	35–17	17–15	21–15	217–157
Minimal change nephrotic syndrome	70	10	8	2	90
Membranous nephropathy	9	26	24	18	77
Focal segmental glomerulosclerosis	5	5	0	8	18
Membranous proliferative glomerulonephritis	1	2	0	4	7
IgA nephropathy	0	6	0	3	9
Mesangial proliferative glomerulonephritis	0	3	0	1	4
Total	85	52	32	36	205

**Fig. 2:**
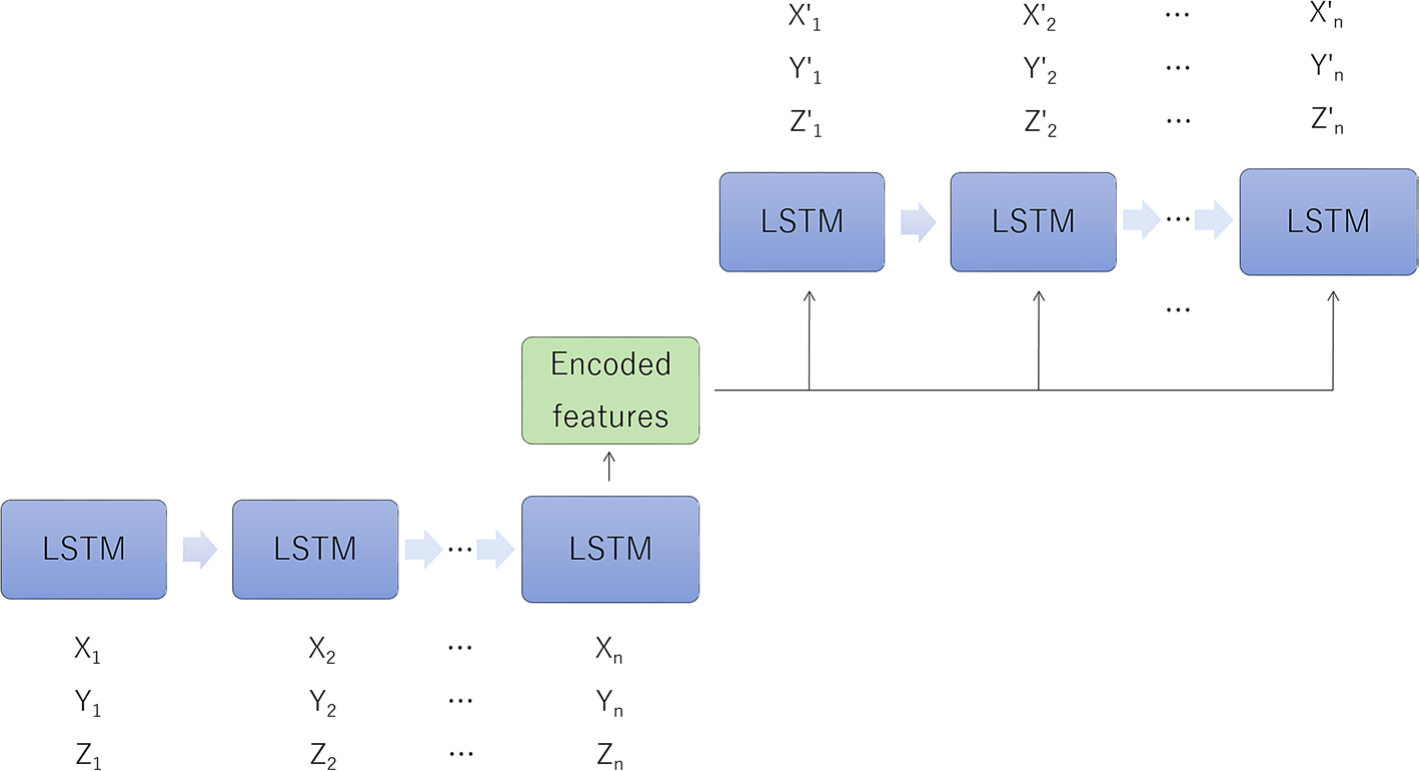
Long short-term memory (LSTM)-encoder–decoder architecture for multiparameter analysis. Input values for three items are shown at n measurement points. In this study, five items and five measurement points were used as input values. Encoded features are regarded to represent the time-series variation patterns of clinical items learned by LSTMs. The features are expressed as a two-dimensional or three-dimensional vector, and can be represented by a scatter plot with one case as one point

C1 through C4 correspond to the clusters classified in Fig. 2

**Table 2:** Blood and urine test values at the time of kidney biopsy for each cluster classified by deep learning

	C1	C2	C3	C4	Total
Creatinine, mg/dL	0.99 (0.57)	1.18 (0.71)	1.20 (0.98)	1.34 (0.79)	1.12 (0.72)
Albumin, g/dL	1.68 (0.64)	2.14 (0.61)	1.94 (0.57)	2.02 (0.58)	1.88 (0.64)
Hematuria, qualitative	1.58 (1.35)	2.37 (1.27)	2.28 (1.17)	2.89 (1.20)	2.08 (1.37)
Proteinuria, qualitative	4.29 (0.84)	4.15 (1.00)	4.48 (0.50)	4.15 (0.59)	4.26 (0.82)
Urine protein, g/gram creatinine	7.51 (3.83)	7.18 (4.23)	8.11 (4.73)	10.25 (6.46)	7.94 (4.63)

C1 through C4 correspond to the clusters classified in Fig. 2. Values, means (SD)

Based on these data, we investigated the clinical time course of nephrotic syndrome. To learn the fluctuation pattern of time-series data consisting of multiple clinical items based on the relationship between measurement points, we utilized the LSTM neural network (Fig. 2). LSTM has an advantage in the modeling of time-series data. LSTM can learn the relationship before and after the measurement point and the relationship between the values of subsequent measurement points. This makes it possible to characterize and classify clinical courses consisting of multiple values and conclusions of doctors. To learn and sort the characteristics of the clinical course, we utilized the autoencoder (self-encoder), an unsupervised machine learning method. An autoencoder is a learning model for dimensional compression whereby the equalization of input and output is optimized. Compressed dimensions are visualized as a scatter plot for the cluster analysis, where formed clusters represent the variations of the clinical course.

**Fig. 3:**
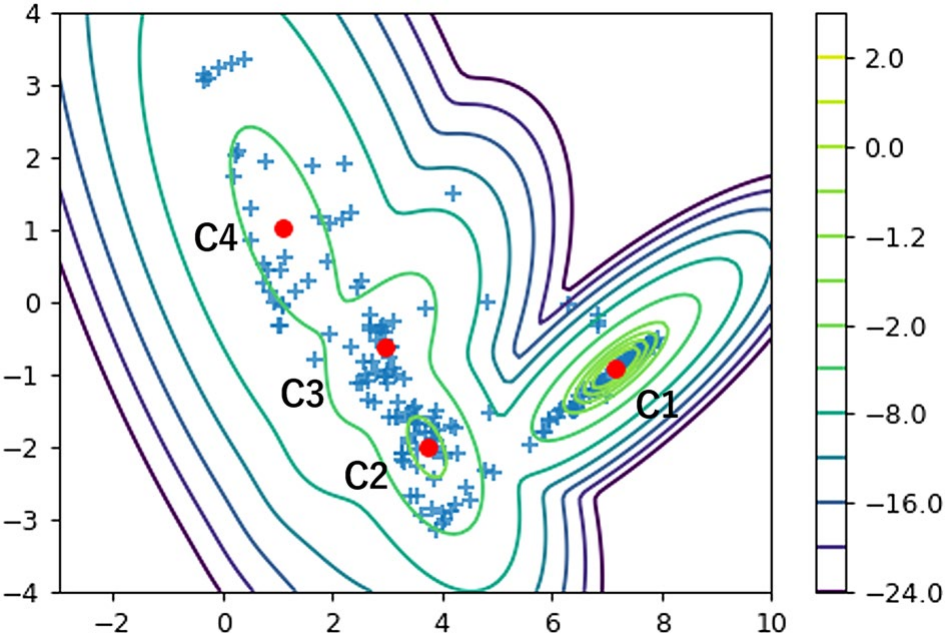
Classification of the natural course of nephrotic syndrome. Using unsupervised machine learning, time-series data of nephrotic syndrome are generally classified into four clusters (C1, C2, C3, and C4). The clusters were defined as Gaussian mixture distributions in a two-dimensional scatter plot (in two-dimensional feature space). The scale bar on the right of the scatter plot shows the log-likelihood. The log-likelihood was obtained over the entire two-dimensional feature space based on the centers (red dots) and variances calculated for the samples, and the contour lines were created by connecting points with the same log-likelihood. Each case is assumed to belong to the cluster with the highest log-likelihood and red dots represent the average vector for each cluster. Based on the initial values of clinical items and the course of each cluster, the severity of disease was considered to increase in order from C1 to C4

The clinical parameters included serum creatinine, serum albumin, dipstick hematuria and proteinuria, and urine protein per creatinine ratio. This selection was based on the following concept; (i) fluctuating items, but not solid ones such as age and sex, which can parallelly change with the clinical course, and (ii) items used to monitor clinical course are selected. Treatment of steroid greatly affects the clinical course of nephrotic syndrome, and a fraction of patients with nephrotic syndrome is treated with steroid before the kidney biopsy. To avoid the treatment bias, analysis was performed using data after kidney biopsy. Both dipstick proteinuria and urine protein per creatinine ratio are included, since they occasionally show the discrepancy [24]. Using unsupervised machine learning, time-series data of nephrotic syndrome are generally classified into four clusters (C1, C2, C3, and C4; Fig. 3). The clusters were defined as Gaussian mixture distributions in a two-dimensional scatter plot (in two-dimensional feature space), and each case was assumed to belong to the cluster with the highest log-likelihood.

**Fig. 4:**
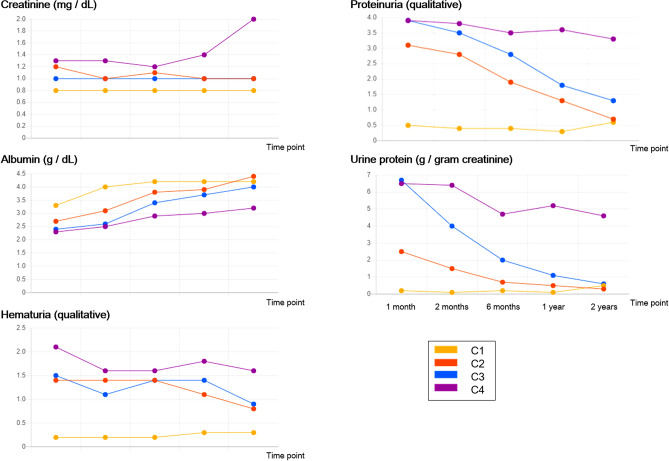
Mean values of each clinical item for each cluster for 205 cases are shown in chronological order. Hematuria and proteinuria are of qualitative values

The mean values of each clinical item for each cluster are shown in chronological order (Fig. 4). Based on the initial values of clinical items and the course of each cluster, the severity of disease was observed to increase in order from C1 to C4 (Figs. 2 and 3). Patients in the C4 cluster showed an increase in serum creatinine in the later part of the follow-up period. Patients in both the C3 and C4 clusters were initially high in both hematuria and proteinuria, whereas the lack of a decline in the urinary protein level preceded the worsening of kidney function in C4.

The distribution of the original kidney diseases in each cluster is shown in Table 1. The largest number of cases was clustered to C1, whose main disease was minimal change disease. The distribution of minimal change disease decreased with the shift of clusters from C1 to C4. Cases of membranous nephropathy were distributed in each cluster. C3 consisted exclusively of minimal change disease and membranous nephropathy. The original diseases of C4 ranged from all the diseases studied in this cohort. An initially and persistently high proteinuria in each disease likely reflected resistance to treatment.

The average blood and urine test values at the time of kidney biopsy for each group are shown in Table 2. The profile was largely indifferent between clusters, suggesting the difficulty of clustering the clinical course at this point in time. Serum creatinine was slightly worsened with the cluster shift from C1 to C4. The most striking difference between C3 and C4 is proteinuria. Although there was no difference in daily urinary protein, the urinary protein per creatinine ratio was worse in C4.

Out of 205 cases, 19 cases were not treated with steroids or immunosuppressive drugs during the observational periods (Fig. 1). Since the reasons for this were uncertain, i.e., very old age and particular prognosis (either good or bad), we performed a sensitivity analysis after excluding these patients. In this analysis, hematuria in the C4 group showed a slightly higher value over the measurement period than in the other groups. In addition, the clinical course of persistent proteinuria followed by actual worsening of kidney function was largely replicated in this analysis (Supplementary Figure S1).

## Discussion

In this study, the LSTM-encoder–decoder architecture classified the clinical course of nephrotic syndrome into four clusters. The classified clusters utilized objective and common clinical parameters, serum creatinine, serum albumin, dipstick hematuria, and proteinuria. The identified clusters showed characteristic clinical courses, which was not necessarily characterized by the original diseases of nephrotic syndrome. These findings suggest the utility of objective clinical parameter-based clustering of patients with nephrotic syndrome to monitor their clinical course and to assess treatment resistance.

This study classified clinical courses according to an unsupervised learning model. Investigation of the changes in clinical items for each classified patient group made it possible to grasp the actual conditions of patients objectively without information related to existing disease classifications. Some of the information obtained is considered rationale, since it is consistent with the medical findings known from conventional epidemiological and case studies, such as the relationship of proteinuria with serum albumin and serum creatinine. Some of the information obtained has not been clarified thus far, such as therapeutic resistance in persistent hematuria. If both hematuria and proteinuria are initially high and the urine protein per creatinine ratio does not decrease as seen in C4, there is a significant decline in renal function. The most striking difference between C3 and C4 is the urinary protein per creatinine ratio, while there is not much difference in daily urinary protein levels. A slightly higher level of protein per creatinine ratio was also observed in C4 patients before kidney biopsy. These results may indicate that the urinary protein per creatinine ratio is more sensitive in clustering the clinical course at the time of kidney biopsy.

The characteristics of each original disease were captured by the cluster. Patients with MN were distributed throughout the clusters, while patients with MCD were mainly distributed in C1. These distributions were in line with the relatively good responsibility to steroids in MCD and the heterogeneous treatment resistance of MN [2], suggesting the validity of the estimation of clinical prognosis based on histopathological diagnosis. On the other hand, the diagnosis of original diseases alone was insufficient to cluster patients with worse prognosis. As represented by C4, a subset of patients with nephrotic syndrome were persistently worse in clinical parameters and showed treatment responsiveness. C4 included almost all types of original diseases. In other words, a certain portion of patients with each disease are likely poor in prognosis.

Based on the patient classification and its characteristics in this study, it is possible to search for new biomarkers related to the worsening and treatment resistance of primary nephrotic syndrome. It is difficult to distinguish between C3, which has a good course, and C4, which shows treatment resistance, based on the test values at the time of diagnosis, and lack of clarity on what kind of background the subject has. For the development of new therapies, it is necessary to investigate the characteristics of the patient group showing treatment resistance more broadly. For example, it is expected that performing whole-exosome analysis of miRNAs [25] or further profiling of kidney biomarkers such as d-amino acids [26–28], long-term undetected enantiomers of amino acids, will not only lead to the discovery of useful novel biomarkers, but also elucidate the mechanism of treatment resistance.

LSTM-autoencoder has an advantage in the classification of time-series information and in the detection of outliers. These features of LSTM-autoencoder have a high affinity in several fields of applied science such as monitoring energy usage status and quality control of industrial product manufacturing processes [12–15]. On the other hand, LSTM has just started to be utilized in analyses of the clinical course. These analyses were commonly performed in a supervised learning setting, for example, classification of diseases such as ICD9 from the test values of multiple measurement points of the target patient [29, 30]. Although supervised learning can support the diagnosis of existing disease classification [31], no effect can be expected beyond clarifying the diagnosis name. Especially in the field of rare and intractable disease, where the correlation between the existing disease classification and the progression of the disease state is not clear, it is necessary to elucidate a new classification of diseases for more a suitable stratification of prognosis. In the case of a disease area where the correlation between the existing disease classification and the progression of the disease state is not clear, no effect can be expected beyond clarifying the diagnosis name. For example, new information cannot be provided for the development of treatments to prevent aggravation. The approach used in this study can provide new information on the development of therapeutics to prevent the aggravation of diseases.

This study has several limitations. The limited number of available patients may prevent a statistically meaningful analysis. Exclusion of patients untraceable at five measured points after kidney biopsy may form a selection bias. The current study classified, but did not predict, the prognosis of patients. Therefore, the features of each cluster could not be extracted in this analysis. For this purpose, novel biomarkers for nephrotic syndrome may be necessary as discussed, which will validate the results of this study.

In conclusion, this study identified four kinds of clinical courses in nephrotic syndrome. This classified clinical course may help objective grasp of the actual condition or treatment resistance of individual patients with nephrotic syndrome.

## Supplementary Information

Below is the link to the electronic supplementary material.

Supplementary file1 (PDF 330 KB)
