# Supplementary material for: Deep learning analysis of clinical course of primary nephrotic syndrome: Japan Nephrotic Syndrome Cohort Study (JNSCS)
Source: Clin Exp Nephrol. 2022 Aug 12;26(12):1170–9. doi: 10.1007/s10157-022-02256-3 (PMC9668942; doi:10.1007/s10157-022-02256-3)
Supplement: Supplementary file 1 — Supplementary file1 (PDF 330 KB) [file 10157_2022_2256_MOESM1_ESM.pdf]

Supplementary Table 1. The number of patients without missing values over 5 measurement points up to 2 years.

Time point	1 month	2 months	6 months	1 year	2 years
Untraceable	4	15	32	48	65
Any missing value	76	61	45	55	51
Traceable with no missing values	294	271	253	227	205

Blood and urine were collected and tested for the following 5 items: serum creatinine, serum albumin, qualitative hematuria, qualitative proteinuria, and urinary protein per creatinine ratio.

Supplementary Table S2. Reasons for untraceability of participants.

Time points	1 month	2 months	6 months	1 year	2 years
Untraceable	4	15	32	48	65
Transfer of hospital	2 (50.0%)	10 (66.7%)	20 (62.5%)	33 (68.8%)	41 (63.1%)
Self-interruption	1	2	2	4	4
Cure	0	0	0	1	3
Ddeath	0	1	8	9	15
Unknown	1	2	2	1	2

Values, numbers of patients (% of all untraceable patients).

Supplementary Table S3. Mean and SD values for Figure 4.

		Creatinine (mg/dL)		Albumin (g/dL)		Proteinuria (qualitative)		Proteinuria (g/gram creatinine)		Hematuria (qualitative)	
Cluster	Time Course	Mean	SD	Mean	SD	Mean	SD	Mean	SD	Mean	SD
C1	1	0.8109	0.2288	3.3348	0.5516	0.4588	0.8339	0.1579	0.2576	0.1529	0.4474
	2	0.7916	0.1765	3.9726	0.5088	0.3529	0.6980	0.1097	0.1654	0.1647	0.6106
	3	0.8114	0.1848	4.1833	0.5789	0.3647	0.8924	0.1838	0.7219	0.2353	0.6970
	4	0.8018	0.2009	4.1901	0.4358	0.2824	0.6618	0.0709	0.2433	0.3059	0.7360
	5	0.8121	0.2038	4.2112	0.6309	0.5529	1.1325	0.5304	2.1263	0.3412	0.7904
C2	1	1.1877	0.9067	2.6860	0.5661	3.0577	1.1505	2.4607	1.6947	1.4038	1.4710
	2	1.0435	0.4079	3.1462	0.5112	2.8462	1.2150	1.5400	1.0861	1.4038	1.4710
	3	1.0598	0.4164	3.7510	0.5006	1.8654	1.1772	0.6794	0.7417	1.4231	1.3636
	4	1.0292	0.3988	3.8810	0.4579	1.2692	1.2877	0.4976	0.8655	1.1154	1.3251
	5	1.0450	0.4131	4.4100	2.6529	0.6538	0.9585	0.3463	0.7212	0.8077	1.0926
C3	1	1.0381	0.3818	2.3747	0.6837	3.8750	1.0232	6.7292	6.9538	1.5313	1.4358
	2	0.9534	0.3152	2.6438	0.6554	3.4688	1.0893	4.0365	2.7696	1.0938	1.0112
	3	0.9628	0.2897	3.3853	0.5892	2.8438	1.2018	2.0322	1.6066	1.4375	1.3679
	4	0.9588	0.2900	3.6950	0.5180	1.8125	1.2854	1.0835	1.2044	1.3750	1.2930
	5	0.9875	0.3723	3.9584	0.5066	1.3125	1.4238	0.6215	0.9350	0.8750	1.2437
C4	1	1.3281	1.1599	2.3000	0.7276	3.9167	0.7592	6.5092	3.2736	2.0833	1.3411
	2	1.2592	1.0107	2.5361	0.6520	3.7500	0.7949	6.3970	3.7161	1.5833	1.5343
	3	1.2067	0.5326	2.8611	0.7350	3.4722	0.8971	4.7472	3.7078	1.6389	1.4748
	4	1.3806	0.6776	3.0428	0.7963	3.5556	1.2121	5.2062	5.0596	1.8056	1.3707
	5	2.0317	2.3425	3.1583	0.8703	3.3333	1.3123	4.5786	3.3294	1.6111	1.3597

Time point 1 to 5 represent 1, 2, 3 months, 1 and 2 years, respectively.

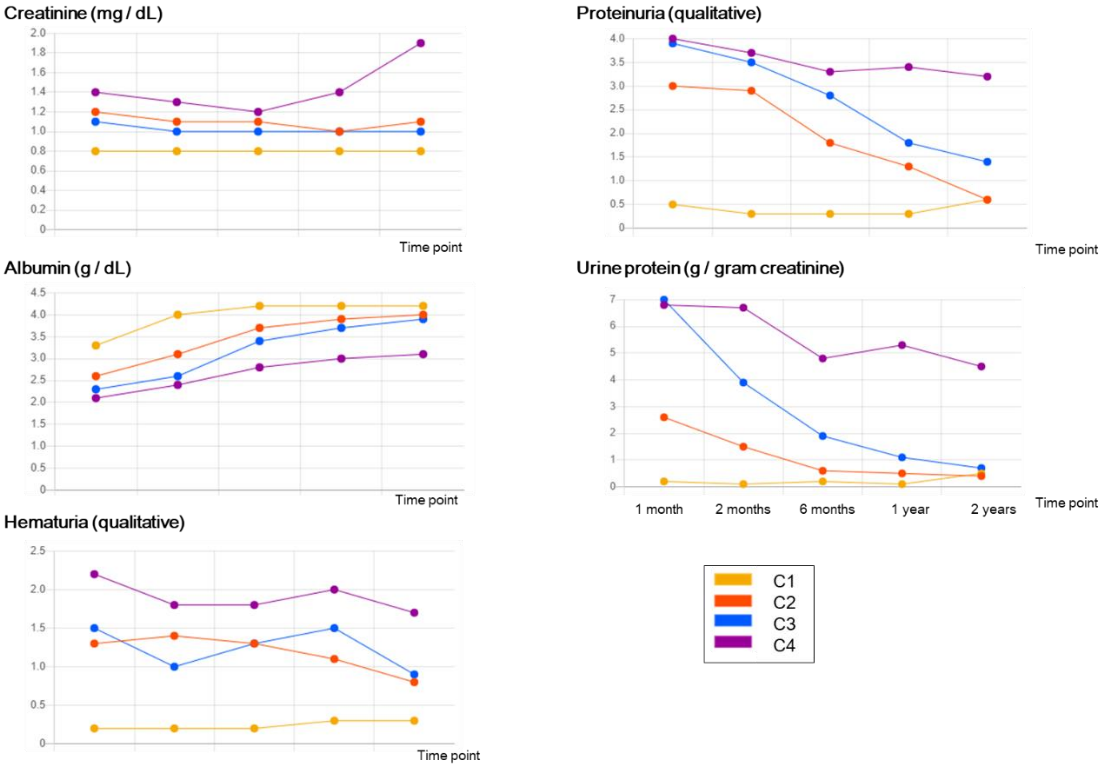

Supplementary Figure 1. Mean values of each clinical item for each cluster for 186 cases are shown in chronological order.
